# A Rare Case of Single Gallbladder and Multiple Pancreatic Metastases of Renal Cell Carcinoma

**DOI:** 10.7759/cureus.31861

**Published:** 2022-11-24

**Authors:** Sana Akhtar, Asma Usman, Anum Sultan, Waleed Khalid, Kashif Siddique

**Affiliations:** 1 Radiology, Shaukat Khanum Memorial Cancer Hospital and Research Centre, Lahore, PAK

**Keywords:** early diagnosis and treatment, contrast enhanced ct, surgery, gall bladder and pancreatic metastasis, renal cell carcinoma

## Abstract

Renal cell carcinoma (RCC) is the most common tumor to metastasize to uncommon sites. Synchronous metastases in the gall bladder and pancreas are rare entities. In this report, we present the case of a 43-year-old male with a complaint of hematuria presenting with a left renal mass. Contrast-enhanced CT revealed an arterially enhancing mass in the left kidney, a synchronous tiny polyp in the gall bladder, and multiple focal lesions in the pancreas. The patient underwent surgery and the tumor was histopathologically labeled as a clear cell RCC with metastases to the pancreas and gall bladder. Post-surgery, the patient has been followed up.

## Introduction

Metastasis of renal cell carcinoma (RCC) spreads in the liver, lungs, bones, adrenal glands, brain, and contralateral kidney. Of note, 30-40% of the RCC cases already have metastatic spread at the time of diagnosis; around 20-50% of the patients who undergo radical nephrectomy develop metastasis later. RCC rarely spreads to the pancreas and gall bladder, with an incidence of 1-3% and 0.6% [1] respectively at post-mortem. In this report, we discuss a case of clear cell RCC with synchronous metastasis to the gallbladder and pancreas.

## Case presentation

**Figure 1:**
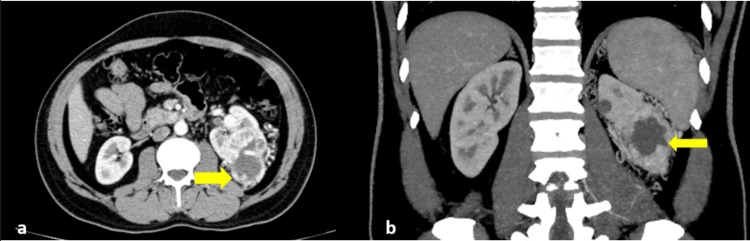
Contrast CT abdomen - axial (a) and coronal (b) images demonstrate enhancing centrally necrotic left renal mass (yellow arrows) CT: computed tomography

The patient was a 43-year-old male who visited a general practitioner with a history of hematuria for two months. CT intravenous urography (IVU) performed at the outside hospital revealed a left-sided renal mass. He was referred to the cancer hospital. His contrast CT demonstrated arterially enhancing centrally necrotic mass involving the interpolar region of the left kidney (Figure 1).

**Figure 2:**
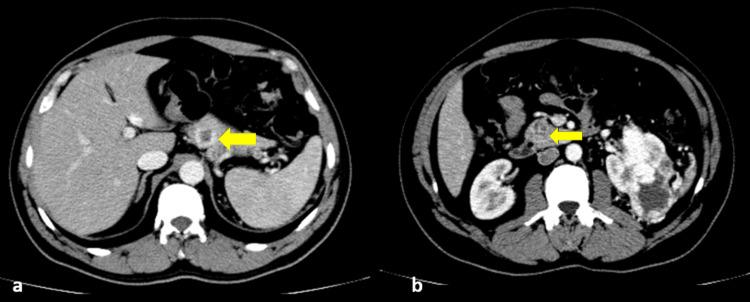
Contrast CT abdomen - axial (a and b) images demonstrate centrally enhancing lesions in the pancreas (yellow arrows) CT: computed tomography

Multiple arterially enhancing centrally necrotic masses in the pancreas, which are of similar morphology as primary renal malignancy, were seen. These were suspected to be multiple pancreatic metastatic deposits (Figure 2).

**Figure 3:**
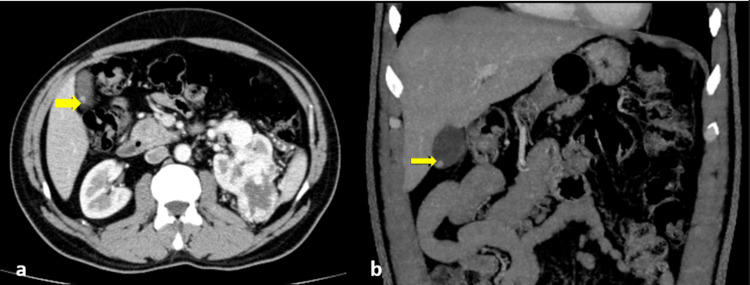
Contrast CT abdomen - axial (a) and coronal (b) images demonstrate a tiny enhancing intraluminal gall bladder lesion (yellow arrows) CT: computed tomography

A tiny avidly enhancing intraluminal focus within the fundus of the gallbladder measuring 4.4 mm, which might represent a polyp, was seen. However, a small mucosal metastatic deposit could not be excluded (Figure 3).

**Figure 4:**
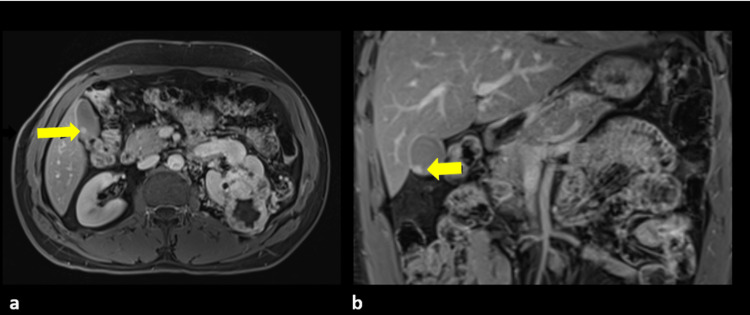
Multiparametric MRI axial (a) and coronal (b) images show an enhancing left renal mass, a tiny enhancing intraluminal gall bladder lesion (yellow arrows) MRI: magnetic resonance imaging

On multiparametric liver MRI, a small enhancing polyp within the gallbladder without pericholecystic infiltration and pancreatic lesions were seen (Figure 4).

Endoscopic ultrasound-guided fine needle aspiration (EUS-FNA) of the pancreatic lesions was performed, which on histopathology showed atypical cells compatible with metastatic RCC.

**Figure 5:**
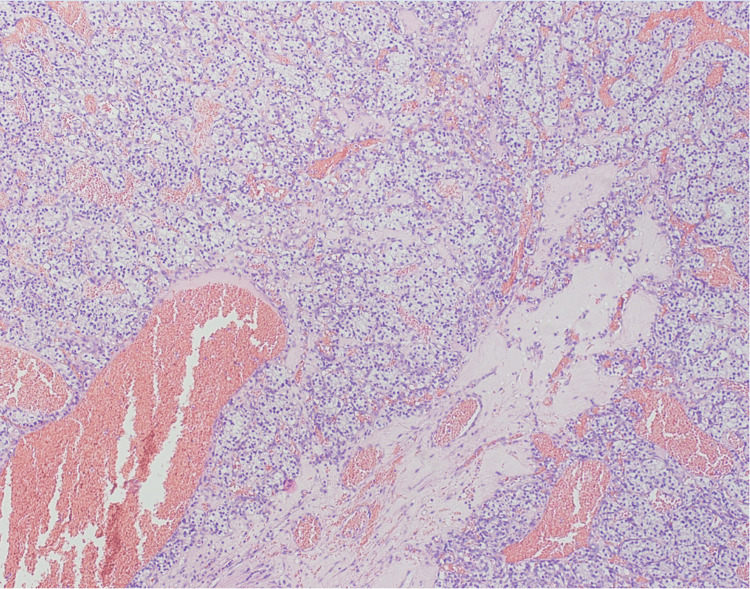
Histological image of renal tumor specimen shows nests of tumor cells with clear cytoplasm and prominent membranes. Prominent arborizing vasculature is also present

**Figure 6:**
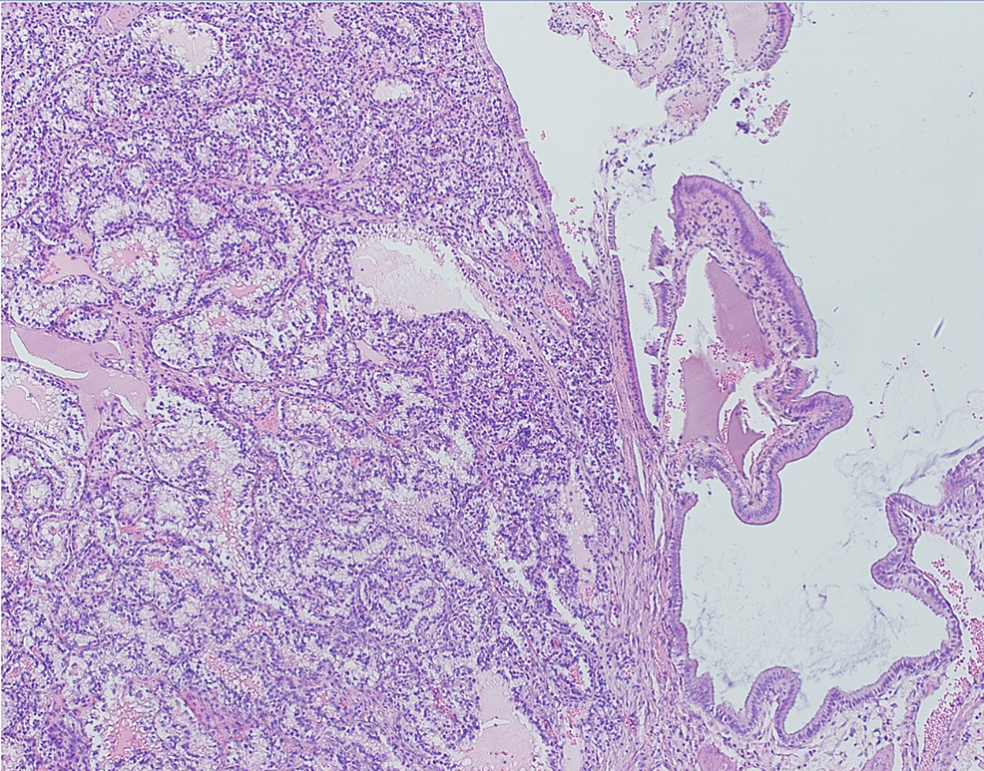
Histological image shows gallbladder wall infiltrated by nests of tumor cells with clear cytoplasm and prominent membranes

**Figure 7:**
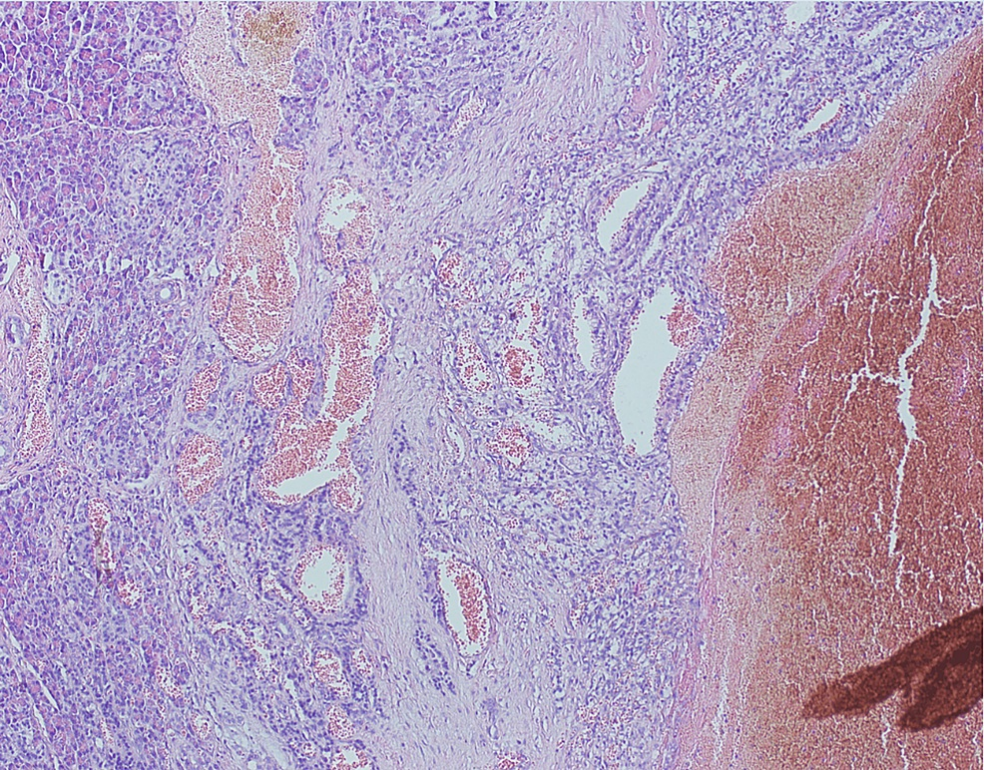
Histological image shows pancreatic parenchyma infiltrated by metastatic clear cell carcinoma

The patient underwent total pancreatectomy, hepaticojejunostomy, gastrojejunostomy, splenectomy, cholecystectomy, and left radical nephrectomy. Histopathological results revealed clear cell RCC (Figure 5). The gallbladder and pancreatic lesions came out to be metastasis (Figures 6, 7). Follow-up observation and imaging were performed after the surgery.

## Discussion

Clear cell RCC metastasizes very commonly. Metastatic spread of RCC most frequently occurs in the lung (75%), followed by bone (20%), hepatic (18%) and adrenal glands (8.9%), brain (8%), and contralateral kidney. The incidence of metastasis to the gallbladder is rare and ranges between 0.4 and 0.58% in the literature [1].

The lion's share of metastasis in the gall bladder is due to melanoma. Lung, kidney, pancreatic, and gastrointestinal cancer metastasis to the gallbladder has also been described [2]. RCC metastasis has some characteristic imaging features and demonstrates avid enhancement. However, differentiating between primary and metastatic gallbladder carcinoma can be challenging. When gallbladder mass is identified simultaneously or metachronously in RCC cases, the possibility of gallbladder metastasis should be considered. Gallbladder metastasis is not indicative of a poor prognosis [1].

Metastatic pancreatic tumors were found in 1-3% of an RCC autopsy series [3]. They represent only about 2% of all pancreatic tumors and are rare pancreatic lesions [4]. There are often multiple when secondary to RCC and reported in 20-45% of cases [5].

Key findings that differentiate secondary pancreatic tumors from primary lesions are multifocality and the pattern of enhancement. RCC metastatic spread to the pancreas has a better prognosis than that of primary pancreatic adenocarcinoma or metastatic lesion of other primaries. Preoperative diagnosis is important as favorable outcomes may be achieved with surgery [6-7].

## Conclusions

This case report discussed a rare incidence of synchronous metastasis of RCC to the gall bladder and pancreas. Metastasis to the gall bladder and pancreas is difficult to diagnose clinically and on imaging. RCC metastasis should be included in the differential diagnosis of RCC patients presenting with enhancing mass on imaging. Early diagnosis followed by resection leads to improved prognosis.
